# Novel software package for cross-platform transcriptome analysis (CPTRA)

**DOI:** 10.1186/1471-2105-10-S11-S16

**Published:** 2009-10-08

**Authors:** Xin Zhou, Zhen Su, R Douglas Sammons, Yanhui Peng, Patrick J Tranel, C Neal Stewart, Joshua S Yuan

**Affiliations:** 1 Institute of Plant Genomics and Biotechnology, Texas A&M University, College Station, TX, USA; 2 State Key Laboratory of Plant Physiology and Biochemistry, College of Biological Sciences, China Agricultural University, Beijing, PR China; 3 Monsanto Co., Saint Louis, MO, USA; 4 Department of Plant Sciences, University of Tennessee, Knoxville, TN, USA; 5 Department of Crop Sciences, University of Illinois, Urbana Champion, IL, USA; 6 Department of Plant Pathology and Microbiology, Texas A&M University, College Station, TX, USA

## Abstract

**Background:**

Next-generation sequencing techniques enable several novel transcriptome profiling approaches. Recent studies indicated that digital gene expression profiling based on short sequence tags has superior performance as compared to other transcriptome analysis platforms including microarrays. However, the transcriptomic analysis with tag-based methods often depends on available genome sequence. The use of tag-based methods in species without genome sequence should be complemented by other methods such as cDNA library sequencing. The combination of different next generation sequencing techniques like 454 pyrosequencing and Illumina Genome Analyzer (Solexa) will enable high-throughput and accurate global gene expression profiling in species with limited genome information. The combination of transcriptome data acquisition methods requires cross-platform transcriptome data analysis platforms, including a new software package for data processing.

**Results:**

Here we presented a software package, CPTRA: **C**ross-**P**latform **TR**anscriptome **A**nalysis, to analyze transcriptome profiling data from separate methods. The software package is available at http://people.tamu.edu/~syuan/cptra/cptra.html. It was applied to the case study of non-target site glyphosate resistance in horseweed; and the data was mined to discover resistance target gene(s). For the software, the input data included a long-read sequence dataset with proper annotation, and a short-read sequence tag dataset for the quantification of transcripts. By combining the two datasets, the software carries out the unique sequence tag identification, tag counting for transcript quantification, and cross-platform sequence matching functions, whereby the short sequence tags can be annotated with a function, level of expression, and Gene Ontology (GO) classification. Multiple sequence search algorithms were implemented and compared. The analysis highlighted the importance of transport genes in glyphosate resistance and identified several candidate genes for down-stream analysis.

**Conclusion:**

CPTRA is a powerful software package for next generation sequencing-based transcriptome profiling in species with limited genome information. According to our case study, the strategy can greatly broaden the application of the next generation sequencing for transcriptome analysis in species without reference genome sequence.

## Introduction

The recent development of next generation sequencing techniques has revolutionized biological and biomedical research and has provided many enabling platforms for systems biology [1,2]. However, maximizing the potential for next generation sequencing heavily depends on available data analysis tools [1]. Some features of next generation sequencing data are different from those of traditional Sanger sequencing. For example, the Illumina Genome Analyzer can generate up to 20 gigabases of short read sequences per run [3]. These short read sequences can be 18 bases, 36 bases or 76 bases in read length. They can also be generated from either single end or paired end runs [4]. The different sequence formats, diverse applications, and the large amount of data generated all require new strategies for sequence analysis [1]. Various sequence analysis tools have been developed to address the needs for different applications of next generation sequencing including *de novo *sequencing, whole genome re-sequencing, metagenome sequencing, transcriptome profiling, microRNA profiling, CHIP-seq, and others [3,5-14]. In this paper, we will focus on a software package providing the enabling tools for cross-platform transcriptome analysis.

Next generation sequencing techniques have enabled several novel approaches for transcriptome profiling [1,4,15]. Depending on the read length, different next generation sequencing techniques can be optimized for different types of transcriptome profiling [16,17]. The 454 pyrosequencing platform provides a longer read lengths of 200 to 400 bases and relatively less sequencing yield at around 200 to 400 megabases per run [18]. Considering the read length, 454 sequencing has some advantages for transcriptome analysis, since the longer reads allow for better assembly of the sequences, which is particularly important for species without reference genome information. As compared to the 454 sequencing, the shorter read length and higher sequencing throughput for SOLiD and Solexa have enabled better transcript quantification, where the deep sequence coverage allows better digital quantification of gene expression levels [16]. Even though not considered as part of next generation sequencing techniques, MPSS (Massively Parallel Signature Sequencing) and iGentifier can also be employed for the semi-quantitative transcriptome profiling with data output similar to the digital gene expression (DGE) profiling [2,3,19]. The so-called digital gene expression profiling technique employs a similar strategy as serial analysis of gene expression (SAGE), in which sequence tags around a four-base restriction enzyme are sequenced and quantified across different samples [20-22]. The SOLiD and Solexa-based methods provide much deeper sequence coverage of the tags and thus provide more accurate quantification. In fact, a recent study has indicated that digital gene expression profiling is more accurate than any microarray platform [21].

Despite the significant advantages of short sequence tag-based gene expression profiling methods, all these platforms, including MPSS, DGE, iGentifier and SAGE are heavily dependent on the availability of reference genome sequence, which limited the application of these techniques to sequenced or well-characterized species only [4,19,23]. However, one of the advantages and tasks for next generation sequencing is to expand the usage of sequence-based transcriptome and genome analysis to a variety of species with limited or no genome information [1]. Novel experimental approaches accompanied by useable software are needed for such analysis.

We hereby describe a new approach for cross platform transcriptome analysis and apply it to a case study. The case study analyzes the molecular mechanisms of herbicide resistance in horseweed, *Conyza canadenisis*, a major weed in US. No genome information is available for horseweed. The project serves as a perfect case study because it uses a strategy to combine different sequencing platforms including 454 sequencing, cDNA sequencing and iGentifier for a comprehensive transcriptome profiling of horseweed's response to glyphosate treatments [24]. The goal of the study was to discover novel genes involved in herbicide detoxification in non-target site resistance, in which multiple pathways including P450, GST and ABC transporters could be involved [24,25]. Limited genome information greatly hinders the application of sequence-based transcriptomic profiling in this and other weedy species [25].

**Figure 1:**
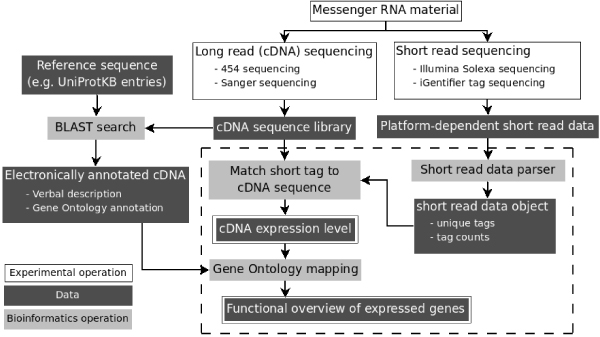
**The schema for the cross-platform transcriptome analysis**. The figure shows how cross-platform transcriptome analysis is carried out. The box within the dashed lines highlights the steps carried out by the CPTRA software package. Basically, the long read sequences are first annotated with blast-based method and then serves as the input for the software. The tag-based sequencing data is another type of input data. The output includes the annotated tags with quantification indicating the level of gene expression and the ontology information.

In order to analyze the aforementioned cross-platform transcriptome analysis dataset, a software package was developed to combine the data from different sequencing techniques to derive the gene expression level, function and ontology annotation. As shown in Figure 1, the data analysis package takes two types of sequencing data including the annotated long-read sequence such as 454 data, and short read sequence tags such as DGE, SAGE, MPSS, and iGentifier. The short sequence tags were parsed to derive a unique set of sequence tags, which could be used for quantification, functional and ontology annotation. The output includes the sequence tag, relative abundance, and the optional GO functional categorization. Considering that the iGentifier data format is similar to that of DGE, we employed the platform to analyze the different datasets from the horseweed study and identified several important candidate genes that might be involved in herbicide resistance.

Herein we present a software package CPTRA for analyzing the transcriptome profiling data from different sequencing platforms. We present software design, data input, and output. The software package is available free at the website: http://people.tamu.edu/~syuan/cptra/cptra.html. We also evaluated the performance of the package and compared the CPU time for different algorithms. In a follow up case study, the software package was employed to analyze our cDNA library-, 454 sequencing-, and iGentifier-data to dissect the mechanisms of non-target herbicide resistance in horseweed. The analysis revealed the effectiveness of the approach for cross platform transcriptome profiling and the potential for the software package to be broadly applied for transcriptome analysis in essentially any species.

## Results

### The software package for cross-platform transcriptome profiling

Figure 1 outlines the schema of the cross-platform transcriptome experimental design and data analysis flow. As shown in the figure, two types of input data are analyzed together. Our previous analysis indicated that the direct annotation of sequences less than 40 bases is not feasible [24]. We therefore developed the CPTRA package with Python for transcriptome analysis based on two or three types of sequencing data. The input of the package is results of sequence tags from different sequencing platforms including DGE and iGentifier, and annotated cDNA sequences of the same species. For the first step of the analysis, the sequence tags are grouped to form a set of unique tags with a count number for each tag. The tags are then aligned to the cDNA sequences under certain limits of allowed mismatch numbers. CPTRA uses the alignment results to compute normalized expression counts for each cDNA sequence.

**Figure 2:**
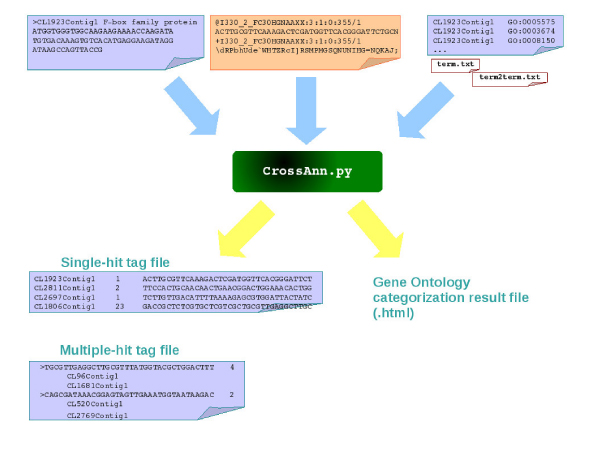
**The input and output data format**. The input format includes both annotated long read sequence and short sequence tags. The output includes the level of expression and the gene function and ontology annotation.

The data input and output formats are as shown in Figure 2. Currently, CPTRA package accepts two types of sequence tag data: Solexa sequencing result in fastq format, and iGentifier sequencing results. The iGentifier technology renders multiple sequence tags of 17 bases, and the Solexa digital gene expression profiling produces multiple sequence tags of around 25 to 35 bases. Both platforms involve sequencing of tags near four base restriction enzyme sites with a similar strategy as SAGE [26]. The platform can thus be expanded for analyzing a variety of different tag-based transcriptome analysis including MPSS and SAGE. The data analysis process is described in the Materials and Methods section with alignment of tag sequence to cDNA reference sequence, and generating expression counts for tags.

**Figure 3:**
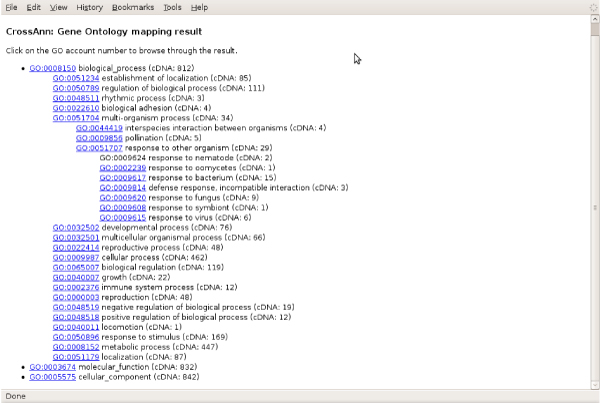
**A snapshot of the GO map as the data output**.

The output files produced by CPTRA operation are as shown in Figures 2 and 3, which include tag alignment result files and GO categorization results (Figure 3). The tag mapping results are composed by three individual files giving detailed conditions on tag mapping output. The output includes the tag information, gene annotation and quantification. The output can be modified to fit into different analysis needs. Besides the sequence tag related output, the GO categorization result will only be produced if the user specifies -G option (See Supplementary_File_2.pdf). The GO categorization result is an HTML file, which can be viewed in a web browser as shown in Figure 3. The maps of cDNA entries to GO terms are displayed as a browsable text tree according to GO vocabulary. Each row is a GO term with its children terms in indented format. The number of cDNA entries is shown to provide a functional overview of the EST/cDNA data. Combined with the tag mapping function, each sequence tag can also be mapped to a certain GO. The CPTRA package will run on any platform with Python interpreter installed (version 2.2 or higher), with no other module or library dependency. No installation is required too. The package can be ran as a standalone program on command line to process the tag data, or used as a programming module to be incorporated into other programs (see online documentation for instructions at http://people.tamu.edu/~syuan/cptra/cptra.html). CPTRA will call Megablast program in NCBI-BLAST package to perform the tag alignment function for Solexa data, therefore, as a prerequisite, the cDNA fasta file has to be formated using formatdb program [27].

### CPU time and performance evaluation

The major speed-limiting step for the CPTRA package lies in the cross-platform sequence matching function. We compared different algorithms including NCBI-BLAST programs (megablast, blastn) and regular pattern search. The regular pattern search basically identifies a string in a sequence file using one of the direct pattern search algorithms implemented with Python. Megablast renders best performance, whilst the regular pattern search is significantly slower than Megablast. The use of regular pattern search is essentially impossible for the large scale of next generation sequencing data, but it allows ambiguity nucleotide code, which is abundant in iGentifier sequencing results. We estimated that processing a dataset with 1000 iGentifier tags and 2500 cDNA sequences will take about 2 hours by the regular sequence search. The NCBI-BLAST programs will take only seconds for such task, but it cannot consider ambiguity nucleotide in alignment. Megablast is thus preferred if the data quality is high and does not have ambiguity nucleotide code.

### Analysis of glyphosate resistant mechanisms in horseweed with the CPTRA package and cross-platform transcriptome profiling

We employed the CPTRA package to analyze the latest sequence-based transcriptome analysis data for our horseweed project [24]. The purpose of the study is to evaluate the effectiveness of the package and the impact of different sequence coverage on the analysis output. The detailed information about the study can be found in our previous work [24]. For this study, there are three types of the input data for the analysis, including the Unigene sequence and iGentifier data as previously presented along with the recent sequenced ESTs with 454 pyrosequencing (Peng, unpublished data) [24]. The iGentifier data is similar to the DGE data and provides short sequence tags for quantification. The 454 EST sequence read length averaged 140 bases and totaled up to 50 megabases for the study (Peng, unpublished data).

**Figure 4:**
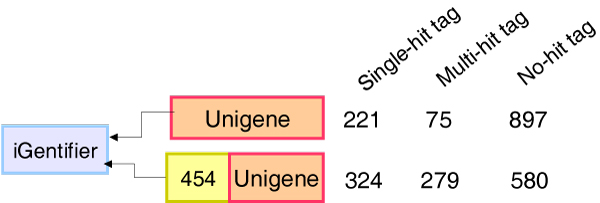
**The performance comparison when different input data are used**. The numbers of the annotated sequence tags were shown for different types input long read sequences. The single hit indicated that each sequence tag only matches one EST. The multiple hit indicated that each sequence tag matches multiple ESTs. The results highlighted that adding 454 EST data greatly improved the performance for cross-platform analysis.

In order to evaluate the effects of the sequence coverage, we first compared the analysis performance based on the different input data when using CPTRA as the analysis platform. The annotated Unigene set was first used as the input and the CPTRA was used to annotate the sequence tags from iGentifier. The analysis lead to the annotation of 296 sequence tags with 221 single hits and 75 multiple hits as shown in Figure 4. We then assembled the EST and 454 sequence data together and used the combined annotated dataset as the input. The outcome yielded more than 600 annotated genes, which doubled the number of annotated sequence tags when using only Unigene as the input. In addition, we have observed more multiple hits as shown in Figure 4. The results highlighted that the number of the sequence tags being annotated will heavily depend on the sequence coverage of the EST or 454 sequencing.

**Figure 5:**
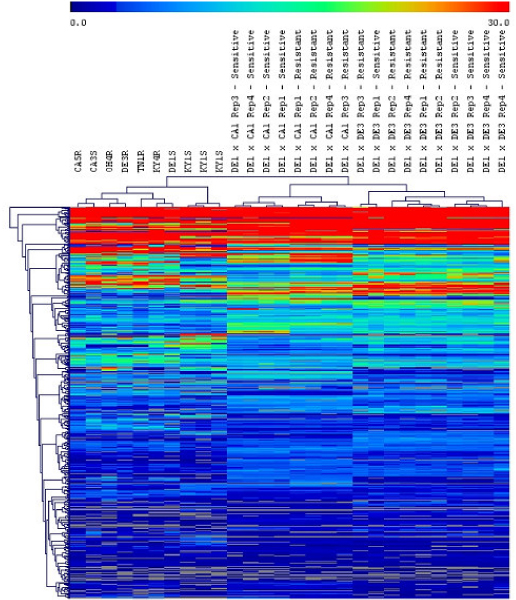
**The cluster analysis of the sequence tags with unique hit and annotation**. The color schema indicates the level of expression. A higher gene expression level is shown with the red color. The samples marked with S at the end are sensitive biotypes and the samples marked with R at the end are resistant biotypes. The short labels indicate the biotypes from different locations in the US, and the long labels indicate the F1 progenies of two different biotypes. For example, DE1XCA1 indicates the plants are from the F1 population of DE1 and CA1 crosses.

Our case study also revealed that the CPTRA package provided the enabling platform for gene discovery through cross platform transcriptome analysis. In order to further our understanding of the molecular and genomic mechanisms of horseweed resistance to glyphosate, we clustered the single hit annotated sequence tags as shown in Figure 5. The samples include both several biotypes from different regions of US and the F1 progeny of some of these biotypes. The F1 progenies were also categorized into resistant and sensitive biotypes based on their resistance toward horseweed. For each cross, multiple replicates of resistant and sensitive biotypes were analyzed by iGentifier. As we can see, the replicates cluster well with one another, indicating the high reproducibility of the data. If the ten biotypes collected from different regions of US were examined, the cluster analysis revealed that the resistant biotypes tend to cluster together and the sensitive biotypes tend to cluster together with the two CA biotypes as the exception. The pattern generally corroborates our previous study [24]. For the F1 progenies, the sensitive and resistant also tends to group.

**Figure 6:**
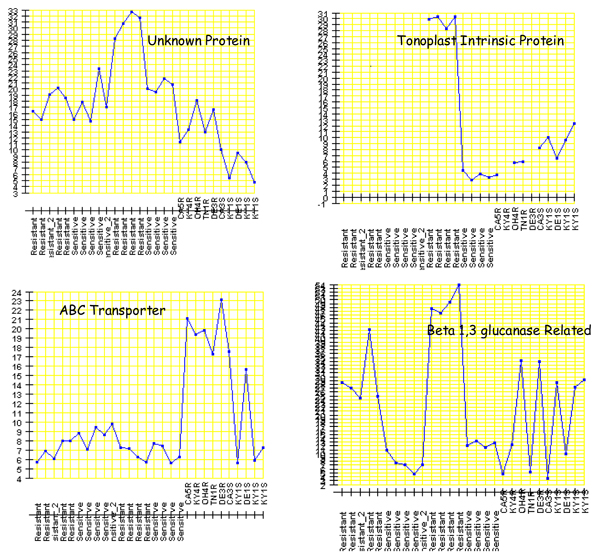
**The expression pattern for several genes significantly up-regulated in some resistant biotypes**. The Y-axis shows the number of the tags, which indicates the expression level. The X-axis shows the different samples, where the samples marked with S at the end are sensitive biotypes and the samples marked with R at the end are resistant biotypes.

The CPTRA analysis allows us to examine the detailed gene expression and function. Basically, the output of expression levels and sequence tag annotation allows us to examine the gene expression pattern of individual genes. The detailed expression patterns for several genes over-expressed in some resistant biotypes were shown in Figure 6. The Y-axis indicated the level of gene expression represented by the count of iGentifier signals. The X-axis showed the different biotypes, indicated by the resistant and sensitive. Several important transporter genes including ABC transporters and tonoplast intrinsic proteins (TIPs) were up-regulated in some resistant horseweed lines, which is consistent with the expectation that cellular transport and sequestration might be important for non-target resistance of horseweed to glyphosate [28-32]. We previously found a TIP gene to be up regulated in some resistant horseweed biotypes. In this study, we identified another TIP gene that was up-regulated in the resistant biotypes [25]. These genes can be candidate genes for down-stream study. Overall, CPTRA provides an effective platform to combine the data from different sequencing techniques to discover novel genes in species without enough genome information.

## Discussion

### Cross-platform transcriptome analysis as a powerful strategy for transcriptome analysis

We have introduced CPTRA as a software package for the cross-platform transcriptome analysis and presented a performance evaluation for CPTRA. The cross-platform transcriptome analysis often involves a short read tag-based platform for transcript quantification and a longer read length platform for annotation. The combination of the two platforms allows us to exploit the advantages for both platforms to reach an accurate quantification and the functional and ontology annotation of the transcripts. Tag-based methods such as DGE, SAGE and iGentifier have been, and will continue to be, broadly applied in global gene expression profiling to provide digital quantification with high confidence [21]. The application of tag-based methods is obviously limited in species without a reference genome or large scale EST data, because the 17 to 50 base sequence tags normally cannot be accurately annotated [16,17]. This limitation requires new complimentary strategies. The recent development of 454 sequencing enables the high-throughput sequence of ESTs with read length up to 400 bases, which can be readily assembled and annotated [16,17]. The combination of long and short read sequencing platforms will allow us to explore the gene expression in a board spectrum of species regardless the available genome information and to quantify the gene expression with the most accurate transcriptome analysis platforms like DGE or SAGE [21,26].

### CPTRA as an enabling tool for cross-platform transcriptome analysis

The software package directly addresses the needs for cross-platform sequence-based transcriptome analysis and provides enablement for next-generation sequencing-based transcriptome analysis. Despite the diverse tools developed for the next generation sequencing analysis, few software packages directly handle cross-platform transcriptome analysis data [1-3,22,33]. The current version of the package allows us to take Solexa and SOLiD short sequence tags along with the iGentifier data as the input for sequence quantification, and to take the annotated 454 or other cDNA sequence data as the source of annotation, thus making the best utilization of short-and long-read sequence data. We will expand the application to MPSS and SAGE in the future. As described in the result part, the software thus provides a comprehensive solution for combined analysis of sequence tags and annotated EST or cDNAs. In order to implement a sequence matching function with a reasonable speed, we also compared different algorithms and determined that the MegaBlast serves as the best option for handling the large dataset generated by the next generation sequencing.

### Comparison of different transcriptome analysis platforms

Global gene expression profiling is a crucial component of functional genomics and the transcriptome analysis tools have been under consistent development [2,22]. Traditional transcriptome analysis platforms include microarray, SAGE, and real-time PCR [21,26,27,34]. The development of next generation sequencing has enabled many novel transcriptome tools, among which sequence tag-based DGE has the promises to become the most accurate option for transcriptome analysis [20,21]. The recent available RNAseq and other methods can also be very powerful in transriptome analysis in species with adequate sequence information [15,17]. However, the performance of RNAseq as compared to the microarray technology has not been well studied as compared to DGE. More importantly, the application of the RNAseq in species with no genome sequence might be difficult because of the complicated assembly of short sequence tags [17]. Even though several new assemblers for short read sequence have been developed, these new assemblers are mostly applied in microbe genome studies currently [1,5,13,35]. The cross-platform global gene expression profiling thus represents a viable choice for transcriptome analysis in species without reference genome because it combines the high accuracy of the DGE and the sequence information from 454 or ESTs. CPTRA provides an enabling software for such analysis.

### Applying cross-platform transcriptome analysis

The case study for the glyphosate resistance data revealed several important considerations for applying CPTRA and cross platform strategies. First, the input sequence coverage is important. As shown in Figure 3, the addition of 454 sequencing data greatly increased the number of annotated tags. Essentially, increasing transcriptome coverage for the input long read sequences serves to provide more tags for annotation. Second, the quality for the sequence assembly is important. Figure 4 shows a significant number of multiple hit annotations. A detailed analysis of these multiple hit annotations indicated that many of these are 454 ESTs have the same functional annotation and these ESTs actually can overlap with one another but failed to meet the assembly criteria. The unassembled 454 singletons can lead to the annotation of the same sequence multiple times. Therefore, both sequence coverage and proper assembly are important for deriving the correct annotation of the sequence tags. Overall, our study showed that CPTRA and cross-platform sequence analysis are powerful solutions for transcriptome analysis in species with limited genome sequence information. The further deep sequencing of the samples with Solexa and more 454 sequencing will allow us to better understand the molecular and genomic mechanisms for the glyphosate resistance in horseweed. The strategy can be used to study a variety of biological questions in many species without reference genome.

## Materials and methods

### Plant growth, RNA extraction, and RNA profiling

All of the plant growth, RNA extraction, sequencing and other bench work were as previously described [24]. The dataset was collected from the published data for further analysis with the CPTRA platform [24]. The sequence assembly was performed using TGICL software http://compbio.dfci.harvard.edu/tgi/software/ with the default settings. The contig sequences were compared with UniProtKB TrEMBL database using blastx program (ncbi-blast package). The annotations including functional description and GO annotation were parsed from the top hit of each contig with an E-value cutoff of 1e-10.

### Software implementation

The software package is implemented in Python. Internally, a major component of the CPTRA package is a class providing universal data handling functionalities, i.e., grouping tags and producing output. The functionalities specific to sequencing platforms were implemented by subclassing. Currently the functions implemented by sub-classing included parsing files with different formats and aligning short read data to cDNA sequences. CPTRA package calls Megablast to align the reads to the reference.

The functional categorization of cDNAs for the annotated ESTs or sequence tags can be obtained based on GO. GO terms should have been assigned to cDNA sequences via previous annotation. Then the software will track each GO term used in annotation to the root term, and meanwhile count how many cDNAs are annotated by each term. According to "true-path-rule", this number would include both the cDNAs directly annotated to the term, and all cDNAs that are annotated by its children terms. Such result is presented in the form of HTML markup language and can be viewed in a web browser that supports JavaScript.

### Horseweed trancriptome analysis

Three types of input data from the glyphosate resistance study have been used. First, a previously published Unigene set was used to perform the cross-platform transcriptome analysis with the iGentifier dataset [24]. Second, the Unigene dataset was assembled together with an unpublished 454 dataset (Peng, unpublished data) and further annotated. The combined dataset was then analyzed together with iGentifer dataset for the cross-platform transcriptome analysis. The single hit tags were then clustered based on the iGentifier expression level. The cluster analysis was carried out with MEV4.0 (Multiple Experiment Viewer), which allowed the output of the cluster results and individual gene expression patterns [36].

## Competing interests

The authors declare that they have no competing interests.

## Authors' contributions

Xin Zhou implemented the CPTRA software under the supervision of Joshua S. Yuan and Zhen Su. Joshua S. Yuan drafted the paper and Xin Zhou and Neal Stewart revised the paper. Doug Sammons, Yanhui Peng, Patrick J. Tranel and Neal Stewart provided the data for the horseweed study.
